# Defining ground truth for prostate segmentation of transrectal ultrasound images: Inter‐ and intra‐observer variability of manual versus semi‐automatic methods

**DOI:** 10.1002/mp.18025

**Published:** 2025-08-11

**Authors:** Louis Lenfant, Clément Beitone, Jocelyne Troccaz, Gaelle Fiard, Bernard Malavaud, Sandrine Voros, Pierre C. Mozer

**Affiliations:** ^1^ Predictive Onco‐Urology, GRC n°5, Sorbonne Université, AP‐HP Hôpital Pitié‐Salpêtrière, Urology Paris France; ^2^ Université Grenoble Alpes, CNRS, INSERM Grenoble INP, TIMC Grenoble France; ^3^ Institut des Systèmes Intelligents et Robotique (ISIR) Sorbonne Université CNRS UMR 7222, INSERM U1150 Paris France; ^4^ Department of Urology Université Grenoble Alpes CNRS, CHU Grenoble Alpes Grenoble INP, TIMC Grenoble France; ^5^ Department of Urology Institut Universitaire du Cancer Toulouse‐Oncopôle Toulouse France

**Keywords:** algorithms, *artificial intelligence, image‐guided biopsy*, image interpretation, computer‐assisted/*methods, imaging, three‐dimensional*/methods, imaging, three‐dimensional/*methods, pattern recognition, automated/*methods, prostatic neoplasms*/diagnostic imaging, reproducibility of results

## Abstract

**Background:**

Accurate prostate segmentation in transrectal ultrasound (TRUS) imaging is essential for diagnosis, treatment planning, and developing artificial intelligence (AI) algorithms. Although manual segmentation is often recommended as the ground truth for AI training, it is time‐consuming, prone to inter‐ and intra‐observer variability, and rarely used in everyday clinical practice. Semi‐automatic methods provide a faster alternative but lack thorough multi‐operator evaluations. Understanding variability in segmentation methods is crucial to defining a reliable reference standard for future AI training.

**Purpose:**

To investigate the inter‐individual variability in manual and semi‐automatic prostate contour segmentation on 3D TRUS images and to compare both approaches to determine the most consistent method that could serve as a reference standard for future AI model development.

**Methods:**

This study is a methodological investigation and not an AI study. Four urology experts independently performed manual and semi‐automatic segmentation on 100 prostate 3D TRUS exams obtained from patients undergoing fusion prostate biopsy. Inter‐individual and intra‐individual variability for manual segmentation was assessed using the Average Surface Distance (ASD) between manually placed points and a reference mesh. Two methods were used to create the reference prostate mesh after manual point positioning: a statistical shape model (manual_SSM) and a deformable model (manual_soft‐SSM). Semi‐automatic segmentations were evaluated using ASD, Dice similarity coefficient, and Hausdorff distance. A Simultaneous Truth and Performance Level Estimation (STAPLE) like consensus method was applied to assess variability across experts in semi‐automatic segmentation. Statistical comparisons used Wilcoxon tests, and effect sizes were calculated using Cohen's d. Bonferroni correction was applied for multiple comparisons. A significance level of *p* < 0.05 (adjusted as needed) was used.

**Results:**

Manual segmentation inter‐individual variability was higher with the manual_SSM method [ASD = 2.6 mm (Inter Quartile Range (IQR) 2.3–3.0)] compared to the manual_soft‐SSM [ASD = 1.5 mm (IQR 1.2–1.8), P < 0.001]. Intra‐individual variability also showed lower ASD values with manual_soft‐SSM compared to manual_SSM, [(1.0 (0.8‐1.1) versus 2.2 (1.9‐2.6), *p* < 0.001], respectively. For semi‐automatic segmentation, inter‐individual variability yielded an ASD of 1.4 mm (IQR 1.1–1.9), Dice of 0.90 (IQR 0.88–0.92), and Hausdorff distance of 5.7 mm (IQR 4.47–7.36). Manual and semi‐automatic segmentation comparisons demonstrated an ASD of 1.43 mm (IQR 1.20–1.90).

**Conclusions:**

The semi‐automatic segmentation method evaluated in this study demonstrated comparable accuracy to manual segmentation while reducing inter‐ and intra‐individual variability. These findings suggest that the tested semi‐automatic approach can serve as a reliable reference standard for AI training in prostate segmentation.

## 1 INTRODUCTION

Accurate and repeatable prostate segmentation of transrectal ultrasound (TRUS) prostate images is crucial for comparing images acquired at multiple time points, multimodal fusion, and prostate‐guided biopsy.^1^ However, several challenges remain in this domain.

First, inter‐reader variability poses a notable issue, compounded by the lack of consensus on the optimal technique for segmenting TRUS prostate images. Manual segmentation is often recommended,^2^ particularly with input from multiple experts, but even this method is subject to variability.^3^ Moreover, manual segmentation can be time‐consuming, which is critical for intraoperative use, such as during biopsy or brachytherapy.

Second, semi‐automatic segmentation methods have been developed, and some are used in everyday practice.^3^ These methods typically require the operator to place key points, after which contours are generated using various mathematical models. These include an algorithm based on a Gabor filter bank,^4^ methods automatically detecting boundary points for edge guidance,^5^ parametric shape modeling,^6^ and spherical harmonics.^7^ The produced contours are typically refined manually with deformable models.^8^

Specifically, Tutar et al. applied spherical harmonics with shape constraints on TRUS images from 29 patients, demonstrating strong alignment with manual segmentation (Mean Absolute Distance (MAD) = 0.40 ± 0.12 mm) but requiring careful initialization by experts.^7^ Gong et al. employed parametric shape modeling with deformable superellipses. On a set of 125 TRUS images from 16 patients, they reported a mean distance between the computer‐generated boundaries and the manual outlines of 1.36 ± 0.58 mm, with some inaccuracies due to individual anatomical variations.^6^ Shen et al. proposed a Gabor filter bank and hierarchical deformation strategy. The comparison of the manual segmentations and the algorithm‐based on eight prostate images yielded an average surface distance (ASD) of 3.20 ± 0.87 pixels with a pixel size of 0.4 mm.^4^ Pathak et al. utilized automatic boundary detection with an anisotropic diffusion filter, resulting in high segmentation consistency across experts on 125 TRUS images from 16 patients (semi‐automatic ASD = 0.7 ± 0.4 mm).^5^ However, manual versus semi‐automatic segmentation comparisons showed greater discrepancies (ASD = 4.0 ± 1.5 mm), suggesting limitations in adapting to individual expert segmentations. The main results from these studies are summarized in Table 1.

**TABLE 1:** Comparison of manual and semi‐automatic prostate TRUS segmentation methods: variability, performance metrics, and effect sizes across studies.

					Manual variability			Semi‐auto variability			
Study	Methodology	Experts	Prostate cases	Images	**Inter‐individual**		Intra‐individual	Inter‐individual	Intra‐individual	Manual vs semi‐auto	Effect size^a^
**Lenfant**	**Manual_SSM**	**4**	477	477	manual_SSM	ASD = 2.6 mm (2.3–3.0) (n = 132)	ASD = 2.2 mm (1.9–2.5) (n = 18)	ASD = 1.4 mm (1.1–1.9) DICE = 0.90 (0.88–0.92) Hausdorff = 5.70 mm (4.47–7.36) (n = 343)	ASD = 1.2 mm (0.9–1.7) (n = 19)	ASD = 1.43 mm (1.20–1.90) (n = 315)	‐
					manual_soft‐SSM	ASD () = 1.5 mm (1.2–1.8) (n = 132)	ASD = 1.0 mm (0.8–1.1) (n = 18)				
Tutar[7]	Spherical harmonics with shape constraints on 3D TRUS images	3	30	30		MAD = 1.34 (±0.66) mm (d = 0.28)^b^	Not evaluated	Not evaluated	Not evaluated	MAD = 1.26 (±0.41) mm, Jaccard = 83.5% (±4.2%)	0.36
Gong[6]	Parametric shape modeling with deformable superellipses and Bayesian segmentation on 2D TRUS images	5	16	594		MAD = 1.82 (±1.44) mm (d = 0.30)^b^	Not evaluated	Not evaluated	Not evaluated	MAD = 1.36 (±0.58) mm	0.13
Shen[4]	Gabor filter bank and Hierarchical Deformation strategy on 2D TRUS images	Unknown	10	10		Not evaluated	Not evaluated	Not evaluated	Not evaluated	ASD = 3.20 (±0.87) mm	−2.47
Pathak[5]	Automatic boundary detection using an Anisotropic Diffusion Filter with Canny's Edge Detector on 2D TRUS images	5	16	125		ASD = 1.8 (±1.4) mm, (d = ‐0.29)^b^ Hausdorff = 4.5 (±2.9) mm	ASD = 3.4±2.2 mm	ASD = 0.7±0.4. Haussedorf 1.8±1.0	ASD = 1.5 ± 0.05mm	ASD = 4.0 (±1.5) mm	−2.29

^a^ Effect sizes (Cohen's d) for the comparisons of Manual versus semi‐automatic variability between the present study and other studies available in the literature.

^b^ Effect sizes (Cohen's d) for the comparisons of inter‐individual variability between the Manual_soft_SSM method of the present study and other studies available in the literature.

Abbreviations: ASD, Average surface distance; SSM: Statistical shape model; TRUS: Transrectal ultrasound.

However, existing literature^4^, ^5^, ^6^, ^7^ mainly focuses on algorithmic descriptions and assessment of semi‐automatic methods with a limited number of patients and experts included, restricting generalizability. Tutar et al. included only 30 patients and three experts, while Gong et al. evaluated 16 patients and five experts without assessing inter‐reader variability of the semi‐automatic method. Shen et al. tested their method on just ten images and also lacked a systematic analysis of inter‐observer reproducibility. These studies primarily describe algorithmic frameworks without multi‐operator validation or clinical integration, leaving key questions about variability and robustness in real‐world settings unanswered. Moreover, there is no comprehensive study evaluating how converting 2D contours or individual points into a 3D mesh influences the segmentation quality. Yet, the transition from 2D annotations or individual points to 3D models is non‐trivial. Differences in slice spacing, interpolation strategy, or mesh‐fitting algorithms can all introduce variation and affect the reliability of the final segmentation. This is a critical gap, as mesh fidelity can have downstream consequences on volume estimation, dose planning, or model training. Despite their increasing adoption in clinical practice, a thorough multi‐operator comparison of manual and semi‐automatic segmentation techniques has yet to be conducted. Additionally, semi‐automated methods still suffer from inter‐reader variability, are time‐consuming, and involve a learning curve.^9^

Last, as the field shifts from semi‐automatic to fully automatic Artificial intelligence (AI)‐driven prostate segmentation,^10^, ^11^ a thorough assessment of existing methods used in daily practice is essential. A variability assessment will help establish the most reliable ground truth for training and validating AI models. The semi‐supervised approach utilized in this study is a shape‐constrained deformable model that incorporates prostate shape priors for TRUS segmentation. It has been implemented in clinical practice for over a decade,^12^ resulting in a substantial database of segmentations performed in real‐world settings using this semi‐automatic algorithm—making it particularly valuable for future AI training. Although this study does not encompass all semi‐supervised learning algorithms, the fundamental principles of this approach (prior‐based deformable models) are widely shared among many semi‐automatic segmentation frameworks,^4^, ^13^, ^14^ ensuring the relevance of these findings to the broader field.

In the context of Magnetic Resonance Imaging (MRI), Molière et al. sought to improve ground truth by quantifying inter‐observer variability in manual prostate contour delineation and determining the optimal number of experts for training.^15^ In the present study, we included four experts, exceeding the minimum of three recommended by Molière et al., to further strengthen the reliability of the reference segmentations.

Similarly, our objective is to quantify inter‐ and intra‐reader variability in manual and semi‐automatic prostate segmentation on 3D TRUS images to determine the most appropriate reference standard for AI training. It has been suggested that the simplest way to demonstrate an algorithm's readiness for clinical application is to show that the differences between its segmentation and those of individual raters fall within the bounds of inter‐rater variability.^16^, ^17^ This highlights the need to evaluate intra‐ and inter‐reader variability for each segmentation method to select the one with the lowest variability.

## 2 MATERIALS AND METHODS

### 2.1 Dataset

A cohort of 100 patients was compiled from the FIONA cohort of 2309 patients who underwent 3D TRUS during a fusion biopsy between January 2016 and July 2021. This dataset included patients fulfilling the inclusion criteria for clinical indication of prostate MRI examination for suspicion of PCa and subsequent targeted transrectal prostate biopsy. From the larger cohort, we selected 100 consecutive patients who underwent prostate biopsy between 2019 and 2021 by the same expert operator in a single center, to ensure high‐quality and consistent image acquisition. No specific inclusion or exclusion criteria were applied regarding prostate size, cancer stage, or other clinical characteristics. This all‐comers design was intended to minimize selection bias and to reflect a representative clinical population. The distribution of prostate volumes in the selected cohort was verified and found to be consistent with that of the broader population. Among the information recorded in FIONA, the 3D TRUS images of the 100 patients were extracted for this study.

### 2.2 3D TRUS protocol

Transrectal prostate biopsy was performed using the Koelis Trinity platform for computer‐assisted fusion of labeled T2‐weighted MRI images with real‐time prostate ultrasound scans, as previously described.^12^ The ultrasound scanning of the prostate was performed automatically using a motorized end‐fire 3D endorectal probe. These transverse TRUS slices were acquired with a 4–9 MHz 3D motorized K3DEC00‐2 TRUS probe. The images had a pixel size of 0.46 mm and a resolution of 256 × 304.

### 2.3 Segmentation randomization

The 100 3D TRUS images were randomly assigned to four independent urology experts from three centers for manual or semi‐automatic segmentation. Each expert was allocated a maximum of 25 prostates for manual segmentation. To ensure a thorough evaluation, a shared subset of more than 10 prostates was manually segmented by all experts to assess inter‐individual variability, and an additional subset of 5 prostates was re‐segmented to assess intra‐individual variability. All four experts were certified urologists with fellowship training. Each had over 5 years of experience performing MRI‐targeted prostate biopsies using the Koelis Trinity platform, ensuring high expertise with the segmentation tasks evaluated in this study.

### 2.4 Segmentation protocol

All operators received standardized instructions on using the user interface for manual and semi‐automatic segmentation. All operators were blinded to each other's segmentations during both the manual and semi‐automatic segmentation processes to ensure unbiased assessment of inter‐observer variability.

For manual segmentation, three reference points (apex, base, posterior) were placed, points were positioned on one medial coronal and sagittal slice, and all subsequent points were positioned on axial slices without any contour display. The expert did not have specific instructions regarding the number of points to position.

**FIGURE 1:**
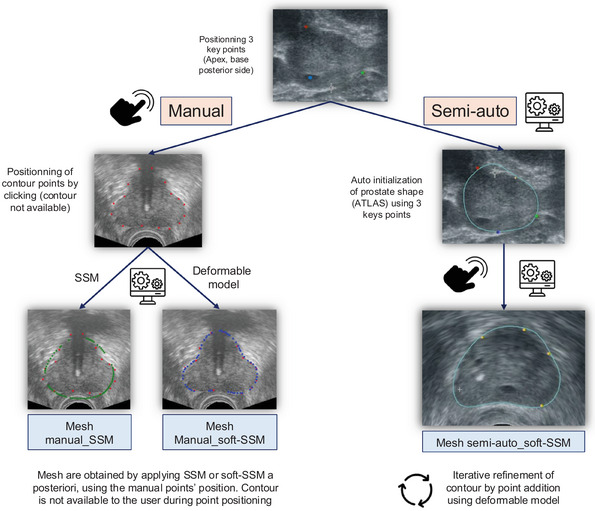
Description of the segmentation methods and comparison. Segmentation was either manual (experts placed points on the prostate contour on the TRUS image without seeing the output of the segmentation) or semi‐automatic (experts placed points with iterative refinement of the prostate segmentation). For manual segmentation, a mesh was obtained a posteriori using either an SSM or a soft‐SSM. Pictograms were sourced from Flaticon and are used under a free license with attribution. SSM, statistical shape model; TRUS, transrectal ultrasound.

In the semi‐automatic segmentation, the same three reference points were placed, after which a mesh was automatically generated using a shape prior.^8^, ^18^ Experts were then free to position points across axial, sagittal, or coronal planes according to their preference, allowing them to adjust the mesh generated by a deformable model (Figure 1).^8^, ^18^ This deformable model incorporates the shape prior, which aims to limit the generation of anatomically improbable shapes. This shape prior is derived from a statistical model of the geometric variability of anatomical structures constructed from a set of prostate segmentations. This shape‐constrained deformable mesh evolves during segmentation under the influence of points placed by the user with interpolation and extrapolation algorithms and is regularized by the statistical model of prostate shapes using B‐Splines. The number of points placed by the expert, and consequently the number of iterations of the shape‐constrained deformable model, was not limited. This method, which produces a final mesh, is referred to as semi‐auto_soft‐SSM and is routinely used on the Trinity platform.

### 2.5 Variability assessment and statistical analysis

#### 2.5.1 Manual segmentation evaluation

For manual segmentations, the points placed on the contour may not be positioned identically, and their number can vary, making direct comparison of point positions an inaccurate reflection of segmentation quality. Instead, we compared the distance between points from one expert to the mesh generated from another or the same expert's manual points. Several methods exist to create the mesh from manual points and may impact the intra‐ and inter‐expert variability. To illustrate this aspect, we used two methods referred to as manual_SSM and manual_soft‐SSM.

Manual_SSM also incorporates a statistical shape model. This model was built using 3D mesh data from 50 patients independent of the study cohort (but also included in the FIONA database). These patients were from the same center, and the same expert acquired their 3D TRUS images as those in the study cohort. The associated meshes were processed to compute the mean prostate shape and principal modes of variation. For this study, given the set of points resulting from the manual segmentation, manual_SSM computed a mesh that strictly adhered to the statistical model and was the closest fit to the points.

The manual‐soft_SSM method utilizes the same deformable as the one used for semi‐auto_soft‐SSM. Compared to manual_SSM, manual_soft‐SSM is more compliant and potentially more robust when handling atypical or underrepresented prostates in the statistical model.

Both manual methods were applied a posteriori after positioning the points in the manual segmentation setting. In other words, the expert did not have access to the mesh or contour estimation when placing the points, meaning that the contour did not influence the point positioning in the manual setting, unlike in the semi‐automatic method.

##### Inter‐individual variability in manual segmentations

**FIGURE 2:**
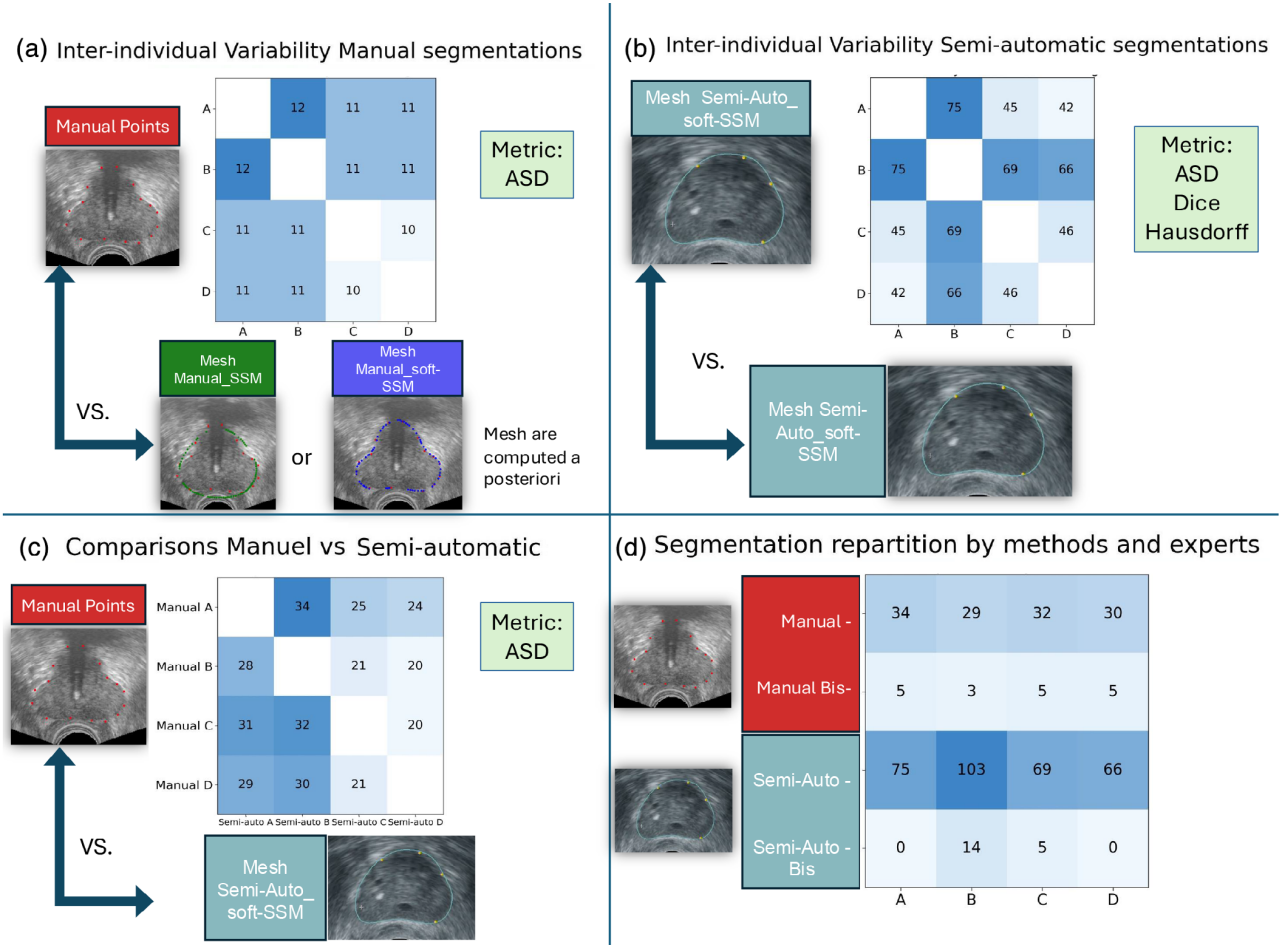
Segmentation comparison and variability assessment. Tables represent the number of segmentations for each pairwise comparison between experts. Bis segmentations represent the segmentation done twice by experts to assess intra‐individual variability. ASD, Average surface distance; DM, deformable model; SSM, Statistical shape model; TRUS, Transrectal ultrasound.

Each expert's manual segmentation was compared with others using the ASD^19^ between the manually positioned points and the mesh generated by either the manual_SSM method or the manual_soft‐SSM method. (Figure 2a).

##### Intra‐Individual variability in manual segmentations

Each expert's repeated manual segmentations of the same prostate were compared using the same ASD measure as in the inter‐individual analysis (Figure 2d).

##### Comparison of the impact of the meshing method on manual segmentation

The impact of the meshing method was assessed by comparing the ASD between manually positioned points and the mesh of the same prostate generated by either the manual_SSM or manual_soft‐SSM method. This comparison helps evaluate the fidelity of the generated mesh concerning the manually positioned points.

#### 2.5.2 Semi‐automatic segmentation evaluation

##### Inter‐individual variability (Pairwise)

Dissimilarity was assessed using the Dice coefficient,^20^, ^21^ a spatial overlap‐based metric that quantifies the similarity between two regions or objects on a scale of 0 (no overlap) to 1 (perfect overlap), Hausdorff distance to assess the maximal distance between two segmentations,^22^ and ASD between each pair of experts. (Figure 2b). Of note, the use of Dice and Hausdorff was possible in this setting because meshes are being compared. The ASD was calculated by measuring the distances from the points of one mesh to the other to determine the ASD.

##### Inter‐individual variability (STAPLE‐like)

Semi‐automatic segmentations were also compared to a consensus of semi‐automatic segmentations from all other experts using the Dice coefficient, Hausdorff distance, and ASD. The objective of the Simultaneous Truth and Performance Level Estimation (STAPLE)‐like approach used here was to average out outliers, thereby producing a consensus that minimizes the effect of individual erroneous segmentations, making it more robust to segmentation errors from specific individuals. The method employed was an expectation‐maximization (EM) algorithm to generate a consensus from 3D meshes by iteratively adjusting the point positions of the computed mesh. In each iteration, the algorithm calculated the distance between each mesh and the current consensus, using these distances to weigh the reliability of each mesh. The consensus was then updated as a weighted average until convergence, focusing on mesh vertices rather than voxel probabilities, as in the traditional STAPLE algorithm.

##### Intra‐individual variability

Differences between repeated semi‐automatic segmentations by the same expert were assessed using Dice, Hausdorff, and ASD.

#### 2.5.3 Manual versus semi‐automatic segmentation comparison

##### Pairwise comparison

The ASD between manual points and another operator's semi‐automatic mesh (Figure 2c), as well as between manual segmentations and the STAPLE consensus, were measured.

##### Comparison to consensus

Manual segmentations were compared to the STAPLE consensus of semi‐automatic segmentation using ASD measurements.

##### Spatial variability

To assess spatial segmentation variability between manual and semi‐automatic approaches, we first assessed point density from manual segmentation. Manual points were registered onto an average prostate model for spatial consistency, revealing regions with varying concentrations of segmented points. ASD values of each registered manual point were visualized using a color scale to highlight areas with higher variability between manual and semi‐automatic segmentation.

The results of inter‐ and intra‐individual variability between manual_SSM and manual_soft‐SSM in manual segmentation were assessed using Shapiro–Wilk tests to evaluate normality. Based on the normality results, Wilcoxon tests were performed to determine significant differences. For all other variability assessments, methods or populations were not directly compared statistically; instead, results were reported with appropriate metrics (ASD, DICE, Hausdorff) using interquartile range (IQR) to describe distribution characteristics.

Effect sizes were computed to quantify the magnitude of differences in segmentation variability between methods. Cohen's d was used as a standardized measure, calculated as the difference between the means of two groups divided by the pooled standard deviation.^23^ When IQR were reported instead of standard deviations, the latter was estimated using the approximation σ ≈ IQR/1.35, assuming a normal distribution. A Cohen's d value of 0.2 was considered a small effect, 0.5 a moderate effect, and 0.8 or greater a large effect, with negative values indicating that the first method in the comparison had lower variability.^24^ These calculations allowed for direct comparisons between manual and semi‐automatic segmentation methods across different studies. Because three main statistical comparisons were performed when assessing segmentation variability across manual methods, a Bonferroni correction was applied to control for the family‐wise error rate.^25^ Adjusted *p*‐values are reported where relevant. Statistical analyses were performed using Python v3.11.9 and R statistical software v.3.4.0 (R Foundation for Statistical Computing, Vienna, Austria).

## 3 RESULTS

Overall, 477 segmentations were performed by four experts: 145 manual and 332 semi‐automatic. The median number of points for manual segmentation ranged between 90 and 496, whereas 39 to 77 points were needed to initiate and iteratively adjust semi‐automatic segmentations. Detailed results are presented in Table 2.

**TABLE 2:** Number of segmentations performed, and number of points positioned for each segmentation modality by operator.

Operator	A	B	C	D
**No. Manual segmentation**	34	29	32	30
**No. Semi‐Auto**	75	103	69	66
**No. *Manual bis***	5	3	5	5
**No. *Semi‐auto bis***	0	14	5	0
**Points manual, No (IQR)**	147.5 (128, 178)	90 (81, 101)	496 (455, 573)	120 (103, 152)
**Points semi‐auto, No (IQR)**	39 (31, 49)	NA	53 (42, 61)	77 (56, 104.5)

Abbreviation: IQR, Interquartile range.

### 3.1 Manual segmentation evaluation

**FIGURE 3:**
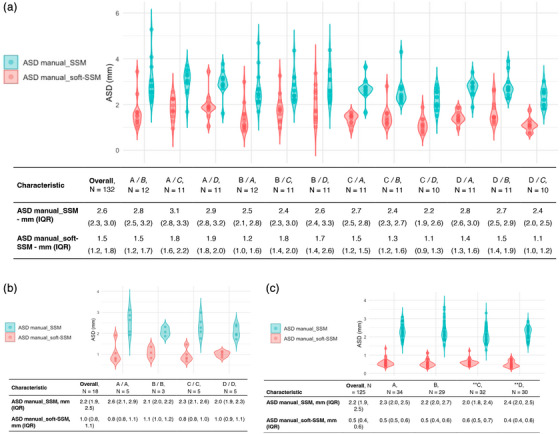
Manual segmentation evaluation. (a). Inter‐individual manual segmentation variability according to the method of MESH creation. (b). Intra‐individual variability of manual segmentation. (c). Intrinsic influence of the method to build MESH from manual points.*ASD: Lower values indicate better agreement between segmentations. ASD, Average surface distance; DM, deformable model; IQR, Interquartile range; SSM, Statistical shape model.

Inter‐individual variability between 132 manual segmentations showed an ASD of 2.6 mm (2.3–3.0) using the manual_SSM and 1.5 mm (1.2–1.8) using the manual_soft‐SSM (adjusted *p* < 0.001) (Figure 3a). For intra‐individual variability, the ASD was 2.2 mm (1.9–2.5) using the manual_SSM and 1.0 mm (0.8–1.1) using manual_soft‐SSM, with 18 comparisons included (adjusted *p* < 0.001) (Figure 3b). The impact of the meshing method evaluated on 125 manual segmentations demonstrated an ASD of 2.2 mm (1.9–2.5) for the manual_SSM method, while the manual_soft‐SSM yielded a significantly lower ASD of 0.5 mm (0.4–0.6) (adjusted *p* < 0.001) (Figure 3c).

### 3.2 Semi‐automatic segmentation evaluation

**FIGURE 4:**
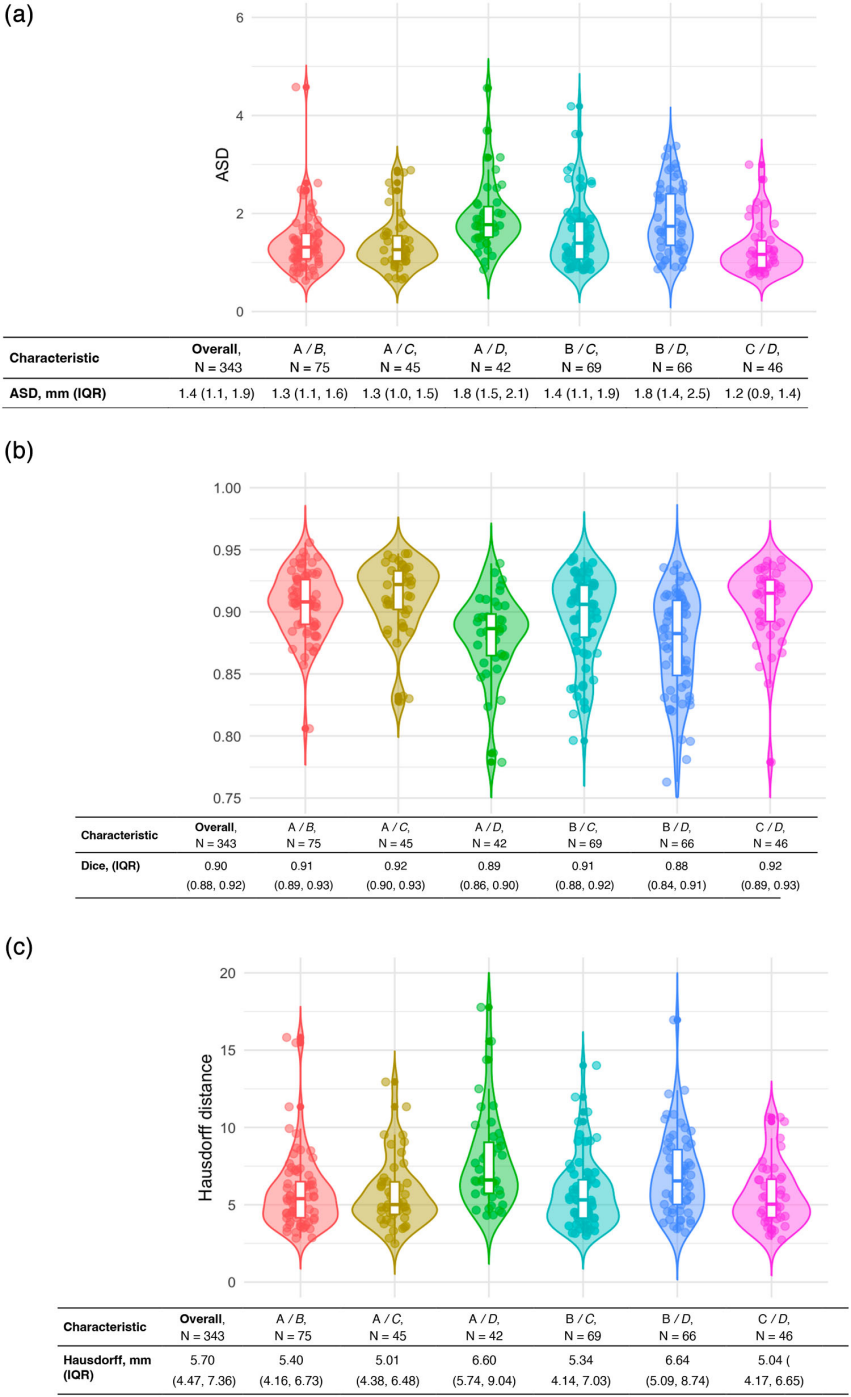
Interindividual variability of Semi‐automatic segmentation in pairwise comparison. (a). ASD. (b). Dice coefficient. (c). Hausdorff distance.*ASD: Lower values indicate better agreement between segmentations. **Dice Similarity Coefficient (Dice): A value of 1 represents perfect overlap between segmentations, meaning higher values are better. ***Hausdorff Distance: Lower values indicate better agreement and reduced segmentation outliers. ASD, Average surface distance; IQR: Interquartile range.

**FIGURE 5:**
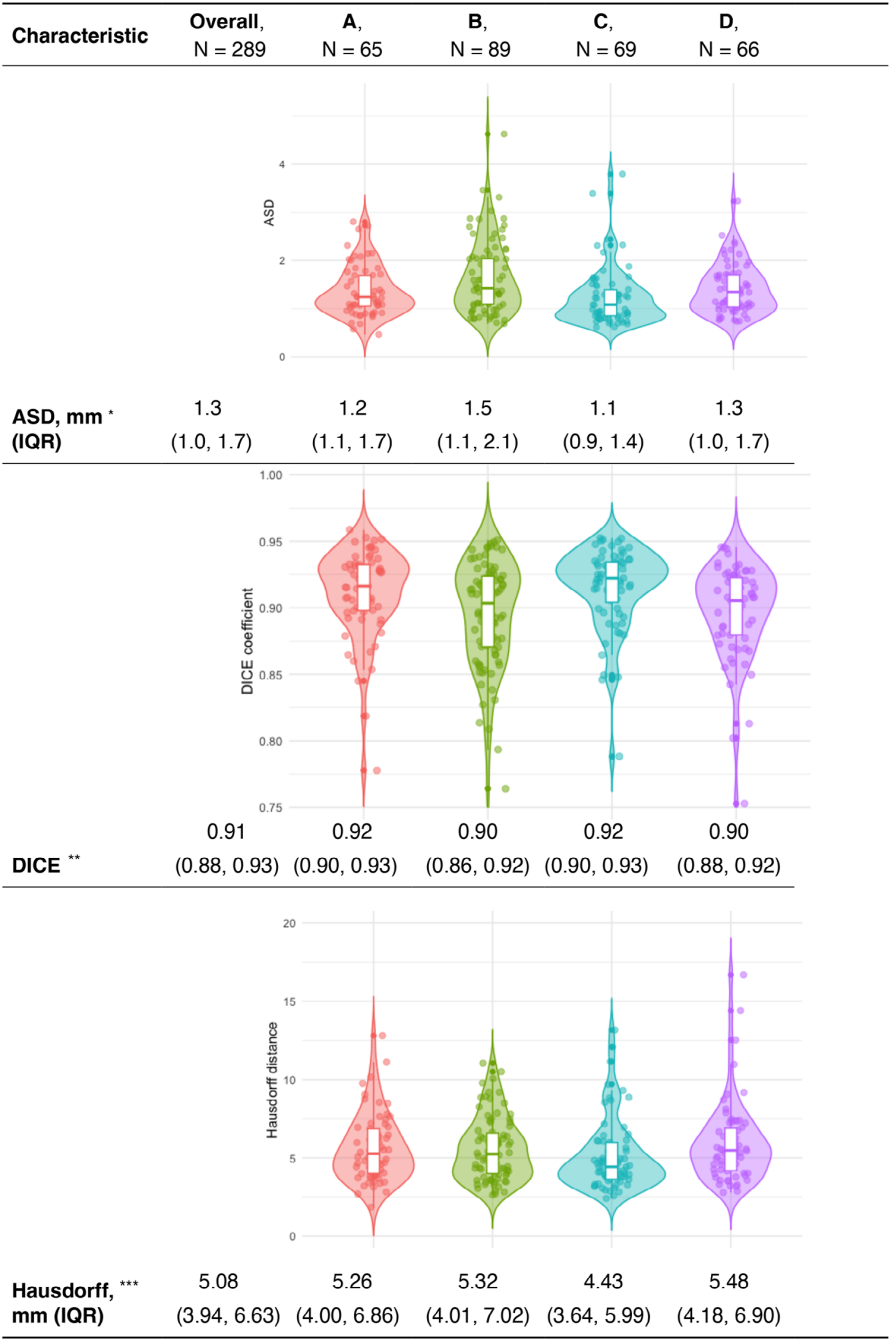
Interindividual variability of Semi‐automatic segmentation compared to consensus obtained via STAPLE. *ASD: Lower values indicate better agreement between segmentations. **Dice Similarity Coefficient (Dice): A value of 1 represents perfect overlap between segmentations, meaning higher values are better. ***Hausdorff Distance: Lower values indicate better agreement and reduced segmentation outliers. ASD, Average surface distance; IQR, Interquartile range.

For inter‐individual variability (pairwise), 343 comparisons yielded an ASD of 1.4 mm (1.1–1.9) (Figure 4a), a Dice coefficient of 0.90 (0.88–0.92) (Figure 4b), and a Hausdorff distance of 5.70 mm (4.47–7.36)(Figure 4c). In the STAPLE‐like approach with 289 comparisons, the ASD was 1.3 mm (1.0–1.7), Dice was 0.91 (0.88–0.93), and Hausdorff was 5.08 mm (3.94–6.63) (Figure 5). Intra‐individual variability based on 19 comparisons from two experts showed an ASD of 1.2 mm (0.9–1.7).

### 3.3 Manual versus Semi‐automatic Segmentation Comparison Results

**FIGURE 6:**
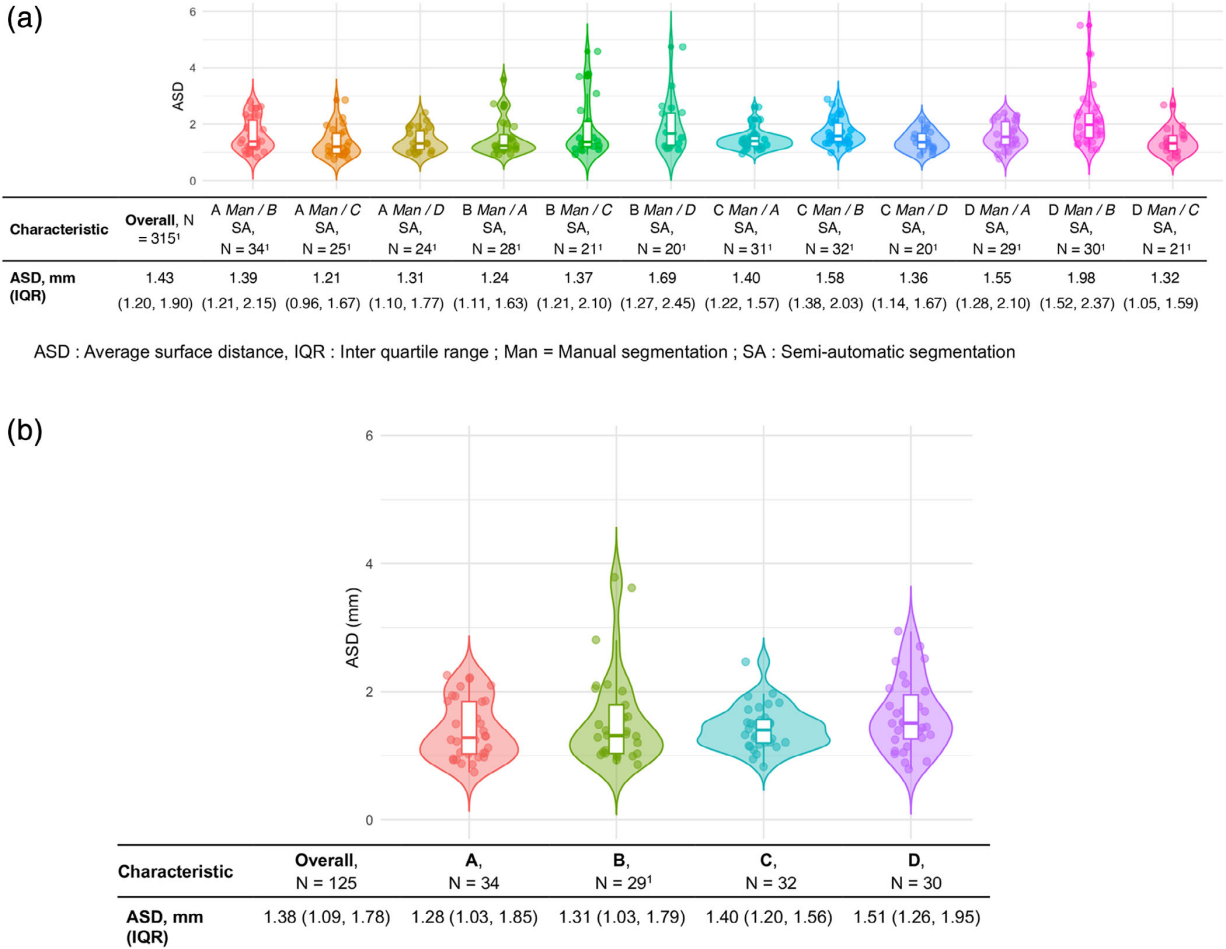
Interindividual variability between manual and Semi‐automatic segmentation. (a). Pairwise comparison. (b). Comparison versus consensus of semi‐automatic segmentation obtained via STAPLE. *ASD: Lower values indicate better agreement between segmentations. ASD, Average surface distance; IQR, Interquartile range; Man, Manual segmentation; SA, Semi‐automatic segmentation.

**FIGURE 7:**
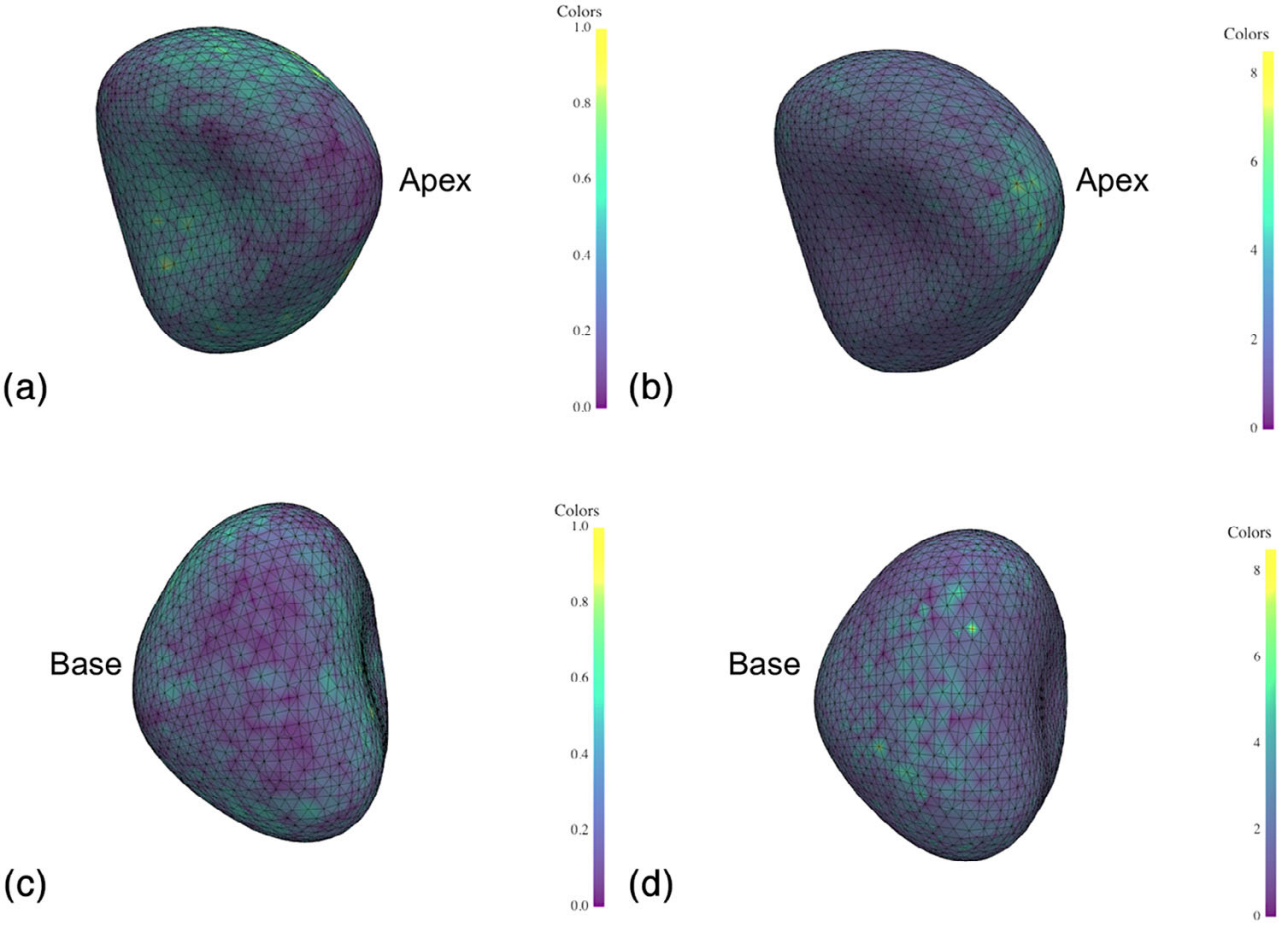
Spatial distribution of the density of manual points and the value of the ASD. (a). Density of points at the apex. (b). Values of ASD at the apex. (c). Density of points at the base. (d). Values of ASD at the base. ASD, Average surface distance.

In the pairwise comparison, 315 comparisons yielded an ASD of 1.43 mm (1.20–1.90) between manual points and another operator's semi‐automatic mesh (Figure 6a). This result demonstrates comparable performance to Tutar (MAD = 1.26 mm, *d* = 0.36) and Gong (MAD = 1.36 mm, *d* = 0.13), with only minor differences in accuracy. In contrast, Shen (ASD = 3.20 mm, *d* = −2.47) and Pathak (ASD = 4.0 mm, *d* = −2.29) show higher variability, highlighting differences in segmentation consistency (Table 1). For the comparison to the consensus of semi‐automatic segmentations, 125 comparisons resulted in an ASD of 1.38 mm (1.09–1.78) (Figure 6b). The evaluation of spatial segmentation variability revealed that the regions with the most ASD variability were localized at the base and apex of the prostate. These areas also correspond to regions with the lowest density of manually positioned points, as visualized in Figure 7. In this figure, panels A and C show the spatial distribution of point density at the apex and base respectively, while panels B and D show the corresponding ASD values. The inverse relationship observed between point density and ASD suggests that reduced manual annotation coverage may contribute to increased variability in segmentation, particularly in anatomically challenging zones like the base and apex.

### 3.4 Effect size analysis

The effect size analysis revealed that the semi‐automatic segmentation method significantly reduced variability compared to manual_SSM (Cohen's *d* = −2.26), indicating a substantial improvement in consistency. In contrast, the difference between semi‐automatic and manual_soft‐SSM was minimal (Cohen's *d* = −0.14), suggesting that both methods achieve similar levels of segmentation reliability. A paired comparison between the manual_SSM and manual_soft‐SSM methods showed a substantial reduction in ASD when using the soft meshing approach. The effect size, calculated using Cohen's d, was 3.01 (95% CI: 2.60–3.40), indicating an extremely large effect and highlighting the marked impact of mesh regularization on reducing segmentation variability.

## 4 DISCUSSION

Prostate segmentation can be performed manually by positioning points or by drawing contours on 2D slices of TRUS images. Regardless of the specific technique, generating a 3D model requires using mathematical algorithms, typically to reconstruct a 3D mesh. This mesh then serves as the ground truth for subsequent comparisons and the training of AI algorithms. In daily urological and radiological practice, semi‐automatic segmentation methods are often preferred due to their efficiency, allowing for interactive adjustments and refinements to the initial contours. This interplay between algorithmic suggestion and human refinement has not been thoroughly studied to date, leaving a gap in understanding how these biases may affect the accuracy, reproducibility, and overall reliability of the final segmentation outcomes. Although studies have been conducted to evaluate the accuracy of manual versus semi‐automatic segmentation methods, they often involve a limited number of patients and images.^4^, ^5^, ^6^, ^7^ Most focus on proof‐of‐concept for semi‐automatic algorithms rather than comprehensive clinical evaluations. Moreover, there is no comprehensive study evaluating how converting 2D contours or individual points into a 3D mesh influences the segmentation quality.

The semi‐automatic segmentation method evaluated in this study corresponds to the one implemented in the Koelis Trinity platform. Although various semi‐automatic algorithms have been described in the literature,^4^, ^5^, ^6^, ^7^ only a few are used in clinical practice.^8^ Among currently available devices, the most commonly adopted strategy consists of a shape‐constrained deformable model initialized by a few anatomical landmarks (e.g., apex, base, posterior border), which is then iteratively refined by the user. This principle is shared by several systems (e.g., Koelis, BK Fusion, UroNav), though specific algorithmic details are generally proprietary and not publicly disclosed. The Koelis system stands out by its integration of this method in routine clinical use and its validation through multiple academic publications,^9^, ^18^, ^26^, ^27^ making it a robust and representative choice for clinical evaluation.

### 4.1 Manual segmentation performance

Our results showed that inter‐individual variability in manual segmentation had an ASD of 2.6 mm (2.3–3.0) when using the manual_SSM (Figure 3a), while using the manual_soft‐SSM yielded a significantly lower ASD of 1.5 mm (1.2–1.8), *p* < 0.001. This finding indicates that the manual_soft‐SSM approach can offer a more consistent segmentation outcome across experts. To further quantify this improvement, an effect size analysis showed a Cohen's d of 3.01 (95% CI: 2.60–3.40) when comparing manual_SSM to manual_soft‐SSM, indicating an extremely large effect and reinforcing the substantial benefit of mesh regularization in reducing segmentation variability. Intra‐individual variability showed comparable results, with an ASD of 2.2 mm (1.9–2.5) using the manual SSM and 1.0 mm (0.8–1.1) using the manual_soft‐SSM, *p* < 0.001 (Figure 3b).

Our results align with previous studies (Table 1). Tutar et al., reported a MAD of 1.34 mm (± 0.66) for inter‐individual variability between three experts in 30 manual segmentations^7^ (Cohen's d = 0.28, relative to the present study's inter‐individual variability of manual segmentation with manual_soft‐SSM), Similarly, Gong et al. found a MAD of 1.82 ± 1.44 mm (Cohen's *d* = 0.30) for inter‐individual variability between five experts for 16 manual segmentations.^6^ Pathak et al reported an ASD of 1.8 ± 1.4 mm (Cohen's *d* = ‐0.29) and a Hausdorff distance of 4.5 ± 2.9 mm for inter‐individual variability between five experts in 16 manual segmentations.^5^ These effect size values (d) indicate the magnitude of differences in inter‐individual variability when compared with our present study's results for manual segmentation using manual_soft‐SSM.

However, in these studies, the method to create a MESH from manual delineation was never mentioned nor assessed. Our study showed that the manual_soft‐SSM method outperformed the manual_SSM method in terms of both inter‐ and intra‐observer variability (*p* < 0.001), providing more consistent results for manual segmentations between experts. Additionally, it showed superior intrinsic accuracy with significantly better alignment between expert‐placed points and the generated meshes.

### 4.2 Semi‐automatic segmentation performance

In the semi‐automatic segmentation evaluation, inter‐individual variability was assessed through pairwise comparisons, yielding an ASD of 1.4 mm (1.1–1.9), a Dice coefficient of 0.90 (0.88–0.92), and a Hausdorff distance of 5.70 mm (4.47–7.36) (Figure 4). These results demonstrate high spatial agreement between experts, indicating robust segmentation performance. The STAPLE‐like consensus method further reduced the ASD to 1.3 mm (1.0–1.7), showcasing the value of generating consensus segmentations in reducing inter‐expert variability (Figure 5).

These findings indicate greater variability than those reported by Pathak et al., the only study evaluating inter‐individual variability for semi‐automatic segmentation.^5^ They reported an ASD of 0.7 mm (± 0.4) for semi‐automatic segmentations (Cohen’*d* = 1.38) and an intra‐expert ASD of 1.5 ± 0.05 mm (Table 1). Our results show higher ASD values, possibly reflecting differences in methodologies and the complexity of our 3D TRUS dataset compared to the 2D TRUS dataset analyzed by Pathak.

### 4.3 Manual versus semi‐automatic comparison

When comparing manual and semi‐automatic segmentations, the ASD between manual points and the semi‐automatic segmentation was 1.43 mm (1.20–1.90) (Figure 6). For manual versus semi‐automatic consensus segmentations, the ASD was 1.38 mm (1.09–1.78) (Figure 5). The effect size analysis revealed that the semi‐automatic segmentation method significantly reduced variability compared to manual_SSM (Cohen's *d* = −2.26), indicating a substantial improvement in consistency. In contrast, the difference between semi‐automatic and manual_soft‐SSM was minimal (Cohen's *d* = −0.14), suggesting that both methods achieve similar levels of segmentation reliability (Table 1). These findings support the use of semi‐automatic segmentation as a robust alternative to manual methods, particularly in reducing inter‐operator variability. This indicates that semi‐automatic segmentation can be reliably used as a ground truth, while manual segmentation is prone to variability depending on the method used to generate a mesh. Additionally, manual segmentation can be time‐consuming due to the large number of points required for accuracy. Semi‐automatic segmentation is particularly advantageous for training AI algorithms as it reduces variability, simplifies data collection from routine segmentations, and decreases the time required for segmentation by reducing the number of points positioned.

Our results for manual versus semi‐automatic inter‐individual variability are comparable to those of Gong et al., who reported a MAD of 1.36 mm ± 0.58 for manual versus semi‐automatic segmentation,^6^ with a small effect size (*d* = 0.13) when compared to the present results for manual versus semi‐automatic segmentation, indicating that the two methods perform very similarly in terms of segmentation consistency. Similarly, Tutar et al., found a MAD of 1.26 mm ± 0.41 with an effect size (*d* = 0.36) relative to the present findings for the same comparison^7^ (Table 1). These similarities highlight the consistency of semi‐automatic methods across different studies, regardless of the specific algorithm employed. However, Pathak reported a higher ASD of 4.0 mm ± 1.5 (Cohen's d = −2.29) for manual versus semi‐automatic segmentations, indicating potential variability across different datasets or segmentation techniques.^5^ Our study's findings on spatial variability are consistent with those of Smith et al., who also identified the apex and base of the prostate as regions with high segmentation variability, particularly in the anterior portions, where the slice increment likely contributed to increased variability.^28^ Smith et al. further noted low variability in posterior regions of the gland in contact with the probe, while lateral and anterior areas exhibited alternating high and low variability, potentially due to differing levels of slice definition. To reduce such spatial variability in future clinical applications, several strategies could be considered. These include optimizing TRUS image acquisition through manual or automatic adjustment of parameters such as focus, gain, depth, and frequency, as well as developing algorithms that account for anatomical variations (e.g., presence of a median lobe or prostate volume stratification). Additionally, the implementation of quality control mechanisms—such as alerting the operator when the number of annotated points is insufficient—may help ensure adequate coverage and improve the reliability of semi‐automatic segmentation tools.

A major strength of our study is its comprehensive analysis of intra‐ and inter‐individual variability in manual and semi‐automatic prostate segmentation methods. Unlike previous studies, which typically involved small cohorts and few experts, we included a diverse dataset of 100 patients with segmentations performed by four experienced urologists from three centers. Regarding the number of experts, we chose to include four from three different centers, as it had been shown that using three independent manual segmentations was reasonable when assessing prostate segmentation algorithms.^15^, ^16^ This design enabled robust multi‐operator comparisons, using standardized statistical metrics, including effect size analysis, to quantify segmentation consistency. In addition to comparing segmentation methods, we evaluated the influence of meshing strategies (manual_SSM vs. manual_soft‐SSM) and the spatial distribution of manually placed points. All segmentations were performed on high‐quality 3D TRUS images acquired within a clinically integrated workflow, enhancing the reproducibility and clinical relevance of our findings.

## 5 CONCLUSION

Our results show that semi‐automatic segmentation methods, particularly those using deformable models based on statistical priors, provide comparable accuracy to manual segmentation while reducing inter‐ and intra‐individual variability. Our study also highlights the benefits of using consensus methods like STAPLE to minimize variability across experts. These insights emphasize the utility of semi‐automatic methods for clinical prostate segmentation, potentially improving the reproducibility and accuracy of prostate cancer diagnosis and treatment planning, but also serving as a reliable ground truth for AI algorithms training.

## CONFLICT OF INTEREST STATEMENT

Pierre Mozer and Jocelyne Troccaz are co‐inventors in the patents for the targeted biopsy device and have been involved in the licensing of the Koelis UroStation system. All other authors have nothing to disclose
